# Association of *PAX3* Gene Polymorphism with Three-Dimensional Nasal Root Morphology

**DOI:** 10.3390/ijms26167842

**Published:** 2025-08-14

**Authors:** Seishiro Ueda, Ryosuke Kimura, Yong-Il Kim, Mohamed Adel, Yu Hikita, Reina Hatanaka, Masahiro Takahashi, So Koizumi, Tetsutaro Yamaguchi

**Affiliations:** 1 Department of Orthodontics, School of Dentistry, Kanagawa Dental University, Yokosuka 238-8580, Japan; s.ueda@kdu.ac.jp (S.U.); hickyyu@gmail.com (Y.H.); hatanaka@kdu.ac.jp (R.H.); takahashi.masahiro@kdu.ac.jp (M.T.); koizumi@kdu.ac.jp (S.K.); 2 Department of Human Biology and Anatomy, Graduate School of Medicine, University of the Ryukyus, Ginowan 903-2720, Japan; rkimura@cs.u-ryukyu.ac.jp; 3 Department of Orthodontics, School of Dentistry, Pusan National University, Yangsan 50612, Republic of Korea; kimyongil@pusan.ac.kr; 4 Department of Orthodontics, College of Dentistry, Texas A&M University, Dallas, TX 75246, USA; mohadel@tamu.edu

**Keywords:** *PAX3*, nasal root morphology, computed tomography, single-nucleotide polymorphism, morphometrics

## Abstract

Paired box gene 3 (*PAX3*) plays an important role in craniofacial development. Mutations in this gene are associated with Waardenburg syndrome, which is a condition characterized by facial anomalies such as widely spaced inner corners of the eyes. *PAX3* gene polymorphisms are associated with the relative position of the nasal root (nasion), even among healthy individuals. Facial morphology has primarily been examined using three-dimensional (3D) facial scans of soft tissues, whereas studies focusing on hard tissues remain limited. Therefore, the present study aimed to analyze 3D craniofacial morphology in hard tissues using computed tomography imaging and investigate the influence of *PAX3* polymorphisms on the 3D morphology of the nasal root. The analysis was conducted on three populations: 201 healthy Japanese, 74 healthy Korean, and 142 healthy Egyptian individuals. DNA was extracted from saliva samples, and the genotypes of two *PAX3* single-nucleotide polymorphisms (SNPs; rs9288572 and rs7559271) were analyzed. A multiple regression analysis of the association between these SNPs and measurements related to nasal root morphology revealed a significant association between rs7559271 and the protrusion angle of the nasion. These findings suggest that *PAX3* gene polymorphisms influence the morphological development of the nasal root within the normal range of hard tissues.

## 1. Introduction

The human face, which is the central site of the primary sensory organs, serves as a focal point for emotional expression and individual recognition. Facial morphology, as a key external defining characteristic, is profoundly involved in self-perception and the formation of interpersonal relationships, significantly influencing psychological and social identity [1,2]. These craniofacial traits have been shaped by natural selection throughout human evolution and developed through a complex interaction between genetic and environmental factors during ontogenesis [3,4]. In recent years, advances in three-dimensional (3D) imaging technologies have enabled the precise capture of subtle differences in facial morphology [5]. These developments have promoted a deeper understanding of individual and population-level variations and clarified the associations between morphology and genetic background [6].

Craniofacial tissues are primarily derived from cranial neural crest cells, and the migration of these cells and the formation of facial prominences require tightly regulated spatial and temporal signaling and gene expression networks [7]. In particular, signaling pathways, such as the fibroblast growth factor (FGF), bone morphogenetic protein (BMP), Sonic hedgehog (Shh), and wingless/Int-1 (Wnt), together with their downstream transcription factors, have been reported to play critical roles in the morphogenesis of craniofacial structures [8,9,10,11]. The paired box gene 3 (*PAX3*) encodes a transcription factor expressed in neural crest cells. It plays a critical role in the development of various tissues, including the cranial nervous system, musculature, and facial skeleton [12]. Mutations in *PAX3* cause Waardenburg syndrome (WS) types I and III, leading to distinct craniofacial features such as an increased intercanthal distance and a rounded or flattened nasal tip [13,14]. *PAX3* mutations have also been reported in craniofacial-deafness-hand syndrome (CDHS), which is characterized by nasal bone hypoplasia or aplasia, hypertelorism, hearing loss, and hand anomalies [15]. Furthermore, developmental studies using mouse models have demonstrated that insufficient *PAX3* suppression expression during the differentiation of neural crest cells impairs osteogenesis and leads to cleft palate formation [16]. These data support the role of *PAX3* as an important regulator of craniofacial bone formation.

Recent genome-wide association studies (GWASs) have demonstrated associations between several single-nucleotide polymorphisms (SNPs) in the *PAX3* region and facial morphology. For example, SNPs such as rs16863422, rs12694574, rs974448, and rs7559271 in *PAX3* are associated with the position of the nasal root (nasion) relative to the eyes [17,18]. These findings suggest that *PAX3* influences craniofacial morphology both in disease states and within the range of normal variation, particularly in the formation of the nasal root area.

Craniofacial morphology is a complex structure composed of hard tissues, including the facial skeleton and overlying soft tissues [19]. However, most previous studies have analyzed 3D facial surfaces based on soft tissues or used two-dimensional head images; relatively few studies have evaluated the associations with the 3D structure of the cranial bone itself. Notably, no associations with *PAX3* have been reported in studies targeting hard tissues in healthy individuals. To better understand the genetic influence on facial morphology, it is essential to clarify the relationship with the underlying hard tissue structures in three dimensions. Furthermore, it is necessary to consider differences in the genetic backgrounds of the target populations when evaluating genetic effects. Allele frequencies and patterns of linkage disequilibrium (LD) vary across human populations, which is thought to reflect their distinct evolutionary histories [20]. Therefore, validating previous findings in different populations is highly valuable and important for understanding the genetic diversity underlying the normal variation in craniofacial morphology.

In the present study, we aimed to analyze 3D craniofacial morphology in hard tissues using cone beam computed tomography (CBCT) and multi-detector computed tomography (MDCT) data in three populations (Japanese, Korean, and Egyptian) and investigated the effects of *PAX3* SNPs on nasal root morphology in healthy individuals.

## 2. Results

**Figure 1:**
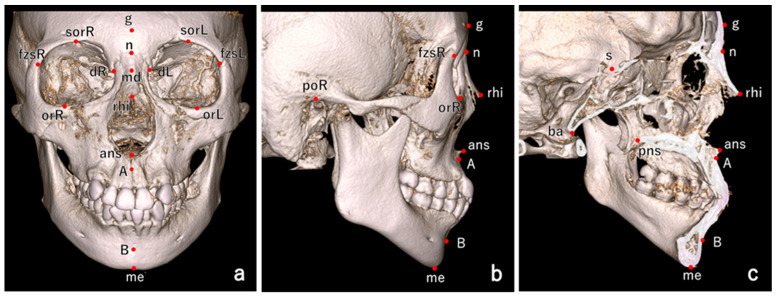
Landmarks used for craniofacial measurements. (**a**) Frontal view. (**b**) Lateral view. (**c**) Clipping-lateral view. Glabella (g); nasion (n); dacryon (d) R/L; supraorbitale (sor) R/L; rhinion (rhi) R/L; orbitale (or)R/L; frontzygomatic suture (fzs) R/L; anterior nasal spine (ans); posterior nasal spine (pns); porion (po) R/L; sella (s); A point (A); B point (B); basion (ba); menton (me); and mid-dacryon (md).

We performed craniofacial morphological measurements by plotting landmarks on CBCT or MDCT images (Figure 1).

The measurement errors in locating cranial landmarks ranged from 0.10 to 0.53 mm (*X*-axis: 0.13–0.50 mm; *Y*-axis: 0.10–0.45 mm; *Z*-axis: 0.10–0.53 mm), confirming high reproducibility.

The craniofacial variables used to evaluate differences among Japanese, Korean, and Egyptian participants, as well as the nasal root measurements, are summarized in Table 1 and Table 2. Cranial width (poR-poL) and height (n-me) were significantly greater in Japanese and Korean individuals than in Egyptian individuals, whereas cranial depth (a-ba) was significantly greater in Egyptian individuals. Moreover, the Egyptian population exhibited significantly smaller nasofrontal angles (g-n-rhi) and shorter nasal bones (n-rhi) than the Japanese and Korean individuals. We detected no significant differences in intercanthal distance (dR-dL) among the three populations, although the anteroposterior height of the nasal root (n-md) tended to be larger among Egyptian individuals.

The results of the analysis of the allele frequencies of the two *PAX3* SNPs in the three populations are presented in Table 3. LD coefficients (D′, r^2^) indicated no strong LD between rs9288572 and rs7559271 (Table 3). The estimated haplotypes are depicted in Table 4.

We performed multiple regression analyses to evaluate the associations between the *PAX3* SNPs (rs9288572 and rs7559271) and nasal root morphology using a single-SNP model, and the results are presented in Table 5. For rs9288572, we observed a significant negative association with the md-n-g angle, which represents the protrusion angle of the nasion, in the Japanese group (*p* = 0.019). For rs7559271, we noted significant negative associations with both the md-n-g angle (*p* = 0.014) and the frontal inclination angle s-n-g (*p* = 0.013) in the Japanese group. In contrast, in the Korean group, rs7559271 was significantly positively associated with the s-n-g angle (*p* = 0.044). In the Egyptian group, we also observed significant negative associations between rs7559271 and both the md-n-g (*p* = 0.0050) and s-n-g angles (*p* = 0.0020). Moreover, a meta-analysis across the three populations revealed significant negative associations for the md-n-g (*p* = 0.0031) and s-n-g angles (*p* = 0.0050). Notably, the association with md-n-g remained significant after a Bonferroni correction (α = 0.05/16). Comparisons of measurement distributions among the genotypes of the two SNPs revealed that in both the Japanese and Egyptian groups, individuals carrying the G allele of rs7559271 exhibited smaller values for both the md-n-g and s-n-g angles (Table 6).

To further examine the associations between the two *PAX3* SNPs and two nasal root measurements, the md-n-g and s-n-g angles, we conducted multiple regression analyses using both a haplotype model and a multi-SNP model.

The haplotype analysis (Table 7) demonstrated that, compared with the ancestral haplotype hGA, the derived haplotype hAG (both SNPs carrying derived alleles) was significantly negatively associated with both the md-n-g (*p* = 0.00055) and s-n-g angles (*p* = 0.015) in the meta-analysis. The haplotype hGG, which carries the derived allele only for rs7559271, was also significantly associated with the md-n-g angle (*p* = 0.0022) in the meta-analysis. In the multi-SNP model incorporating both rs9288572 and rs7559271 (Table 8), rs7559271 remained significantly associated with the md-n-g and s-n-g angles, whereas rs9288572 exhibited no significant association. Moreover, we detected no significant interaction between the two SNPs.

**Figure 2:**
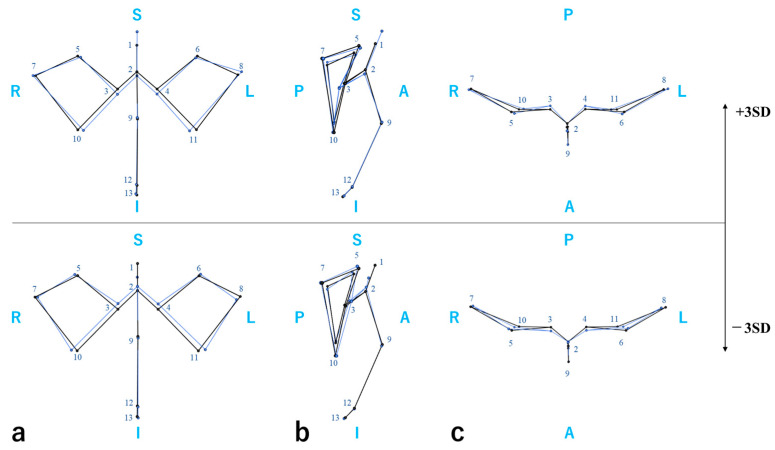
Shape variation along PC5 is visualized as deviations of ±3 SD from the mean 3D coordinates. (**a**) Frontal view. (**b**) Lateral view. (**c**) Top view. S—superior, I—inferior, A—anterior, P—posterior, R—right, and L—left. Black lines represent the average configuration, while blue lines represent the variation in interest. 1. Glabella; 2. nasion; 3. right dacryon; 4. left dacryon; 5. right supraorbitale; 6. left supraorbitale; 7. right frontzygomatic suture; 8. left frontzygomatic suture; 9. rhinion; 10. right orbitale; 11. left orbitale; 12. anterior nasal spine; and 13. A point.

To assess structural variation in the upper facial shape including the nasal root, we performed a generalized Procrustes analysis (GPA) and principal component analysis (PCA) based on upper facial landmarks (Figure S1). The cumulative contribution of the top five principal components (PCs) was 63.9%. PC1 represented the anteroposterior projection and depression of the upper face, PC2 the vertical dimension of the upper face, PC3 the nasal bone length, PC4 the anteroposterior contour of the orbital region, and PC5 the anteroposterior projection and depression of the glabella and dacryon. A regression analysis for the PC scores indicated that rs7559271 was associated with PC1 in Korean individuals (*p* = 0.016) and with PC5 (Figure 2) in Japanese individuals (*p* = 0.048) and Egyptian individuals (*p* = 0.029). However, the associations with PC5 exhibited opposite directions, causing the meta-analysis to lose significance (Table 9).

## 3. Discussion

In the present study, we investigated the effects of two *PAX3* SNPs, rs9288572 and rs7559271, on craniofacial morphology, focusing on the nasal root, based on 3D cranial structures in Japanese, Korean, and Egyptian populations. Multiple regression analyses using nasal root-related measurements revealed significant negative associations between rs7559271 and both the nasion protrusion (md-n-g angle) and frontal inclination angles (s-n-g angle) in the meta-analysis across the three populations. Notably, the association with the md-n-g angle remained significant even following a Bonferroni correction. These two measurements exhibited significant negative associations in both the Japanese and Egyptian populations. Decreases in the nasal root protrusion and frontal inclination angles reflect the anterior displacement of the nasal root and posterior inclination of the forehead. Furthermore, these angles are susceptible to the vertical position of the glabella; a higher glabellar position may be accompanied by a reduction in the supraorbital prominence, which can result in smaller angles. Therefore, these results suggest that *PAX3* polymorphisms contribute to morphological changes characterized by nasal root flattening.

Several SNPs in *PAX3* associated with facial morphology have been reported in previous GWAS [6,17,18,21,22,23,24,25,26,27]. Paternoster et al. [17] demonstrated that rs7559271, located in an intronic region of *PAX3*, is associated with the 3D distance between the nasion and the midendocanthion. They further analyzed the directionality of this distance and reported that the YZ-plane components (vertical and anteroposterior directions) mainly contributed to the association. Liu et al. [18] reported that rs974448, located approximately 60 kb downstream of *PAX3*, is associated with the distance between the nasion and the center of the eyeball. Moreover, Bonfante et al. [25] demonstrated that rs10176525 is related to the nasal root depth and nasion position, while Zhang et al. [26] found that rs9288572 is associated with the segmented nasal region of the face. Although rs9288572 is located within 80 kb of rs974448 and rs10176525, it resides in a different LD block. In contrast, rs7559271 and rs974448 are located within the same LD block. SNP candidates with extremely low minor allele frequencies in the study populations were excluded from the present analysis due to the limited statistical power to detect associations. Based on these previous findings, we selected rs9288572 and rs7559271 as the target SNPs for the present study.

*PAX3* is a key transcription factor involved in neural crest cell differentiation and migration, as well as in osteogenesis, and has been implicated in syndromes such as WS and CDHS [12,13,14,15]. Previous studies have suggested that *PAX3* affects not only disease phenotypes but also facial variations within the normal range in healthy individuals. In the present study, we also confirmed an association with the md-n-g angle, which reflects the YZ-plane direction from the mid-dacryon (corresponding to the midendocanthion) to the nasion, consistent with the findings of Paternoster et al. [17]. Although an increased intercanthal distance is a hallmark of WS and other syndromes involving *PAX3* mutations, we observed no significant differences for angles representing intercanthal width (dR-s-dL and dR-n-dL angles). These findings suggest that craniofacial variations associated with *PAX3* polymorphisms in healthy individuals occur within the normal range and differ from the phenotypic alterations observed in disease conditions.

In the present study, we observed a significant association between rs7559271 and the md-n-g angle, whereas rs9288572 exhibited only a suggestive association with the md-n-g angle in the Japanese population and no significant associations with other traits or in other populations. These differences may reflect variations in the genetic background and LD structure among populations. Consistent with this, we did not observe a strong LD between rs9288572 and rs7559271, suggesting that the two SNPs may be involved in distinct regulatory regions. Therefore, we conducted a multi-SNP model analysis incorporating both SNPs as explanatory variables. In the meta-analysis, rs7559271 demonstrated significant negative associations with the md-n-g and s-n-g angles, whereas rs9288572 did not exhibit any significant associations. Moreover, we noted no significant interaction effect between the two SNPs. A haplotype analysis further revealed that hAG, a haplotype carrying the derived alleles of both SNPs, exhibited significant negative associations with multiple angles related to nasion protrusion (md-n-g angle, s-n-g angle). The findings of these single-SNP and haplotype analyses support the notion that rs7559271 is involved in the degree of nasion protrusion and may influence the 3D structure of the nasal root, including frontal and medial orbital convexity or flattening. Although rs9288572 did not exhibit significant associations in the single-SNP model, its potential effect emerged in the haplotype-based analysis. This suggests that the cumulative effects of multiple SNPs may contribute to the association. Notably, for complex traits such as facial morphology, both single SNP effects and epistatic interactions among multiple genes can play crucial roles in shaping morphological features [28]. Furthermore, non-coding regions, which account for most of the genome, harbor many functional elements and polymorphisms associated with disease and traits. These regions form complex regulatory networks through interactions both near and distant from the loci [29,30]. Although we detected no clear interaction between the two SNPs in this study, further analysis of interactions among polymorphisms both within and outside the *PAX3* region may enhance our understanding of the genetic regulatory networks underlying facial morphology.

The inter-population differences in associations with nasal root morphology observed in this study may be influenced by both genetic and environmental factors. Allele frequencies and LD patterns differ among populations, and the phenotypic effects of the same SNP may vary across populations [31]. The nasal root landmark, nasion, is located on the frontonasal suture, where the frontal and nasal bones articulate. This suture is a dynamic growth site involved in the remodeling of the upper face. Animal studies have reported that mechanical stimuli can influence bone formation and remodeling of the nasal and frontal bones via the frontonasal suture [32]. Furthermore, craniofacial sutures are sites of active bone formation and resorption, integrating mechanical loading and molecular signaling to regulate local morphology, such as the growth direction of the surrounding bones [33,34]. Therefore, the inter-population differences in associations with nasal root morphology observed in this study may be attributable not only to genetic influences arising from LD patterns but also to mechanical factors related to morphological differences in other craniofacial regions, such as jaw morphology.

Previous research on the relationship between *PAX3* and facial morphology has mainly focused on soft tissues using 3D facial scans or magnetic resonance imaging, and no associations with *PAX3* have been reported in studies targeting hard tissues in healthy individuals. Moreover, regions such as the nasal root and forehead, which have demonstrated associations in prior studies, are covered by thin, soft tissue and are strongly influenced by the underlying skeletal structure [35,36]. A key feature of the present study is the direct 3D evaluation of the facial skeleton using high-resolution CBCT and MDCT images, independent of the overlying soft tissue surface.

Nevertheless, the present study had certain limitations. The two SNPs analyzed in this study, rs9288572 and rs7559271, are located within LD blocks that encompass most of the *PAX3* SNPs previously reported to be associated with nasal root morphology. However, due to constraints in sample size and minor allele frequencies, the SNPs evaluated were limited to these two, and the *PAX3* gene region and its surrounding genomic areas were not comprehensively covered. Furthermore, the overall sample size was relatively small, and the study populations were unevenly distributed, with the number of Korean participants being approximately half that of the Japanese and Egyptian groups. This limited sample size in the Korean group may explain the lack of significant findings in this population.

Therefore, future studies should increase the sample size and conduct analyses across multiple populations, including additional SNPs both within and around the *PAX3* region. Moreover, enhancer activity during facial development changes dynamically throughout different stages of craniofacial morphogenesis [37]. Therefore, functional annotation analyses using epigenomic databases will be essential to investigate whether the target SNPs are involved in regulatory regions or transcription factor binding sites relevant to facial development.

The nasal root is a key region that contributes to facial appearance [38] and holds the potential for advancing facial prediction and guiding treatment planning in orthodontic and plastic surgery fields. As demonstrated in mouse models, differences in the timing of *PAX3* expression can affect bone growth [16], suggesting that even subtle polymorphisms may influence morphogenesis. This study contributes to our understanding of the genetic basis underlying normal variations in craniofacial morphology.

## 4. Materials and Methods

### 4.1. Data Collection

Craniofacial data and genomic DNA were collected from 201 Japanese, 74 Korean, and 142 Egyptian individuals. The Japanese participants included 56 men (aged 18–55 years; mean age, 25.9 years) and 145 women (aged 18–65 years; mean age, 27.0 years). The Korean participants consisted of 29 men (aged 18–44 years; mean age, 24.7 years) and 45 women (aged 18–47 years; mean age, 26.3 years). The Egyptian participants included 63 men (aged 18–37 years; mean age, 23.8 years) and 79 women (aged 18–50 years; mean age, 23.8 years). All participants, including those from Japan, Korea, and Egypt, underwent CBCT or MDCT for diagnostic purposes at their respective orthodontic clinics. Individuals with congenital anomalies such as a cleft lip and palate or with systemic diseases were excluded. Only healthy individuals aged 18 years or older were included in the study. Written informed consent was obtained from all participants prior to their inclusion in the study. This study was conducted in accordance with the guidelines of the Declaration of Helsinki and was approved by the relevant ethics committees at each participating institution: Kanagawa Dental University (No. 955), Pusan National University (IRB No. PNUDH-2019-025), and Suez Canal University (IRB No. 8).

### 4.2. Genotyping

Saliva samples were collected from participants using the Oragene DNA collection kit (DNA Genotek, Ottawa, ON, Canada), and genomic DNA was extracted from the saliva samples. Genotyping of the *PAX3* SNPs rs9288572 and rs7559271 was performed using a TaqMan genotyping assay (Life Technologies, Carlsbad, CA, USA) [39]. Allele frequencies were determined from the obtained genotypes.

Haplotypes were inferred using PHASE version 2.1.1 [40]. LD coefficients (D′ and r^2^) were calculated using Haploview version 4.1 [41] (Table 3 and Table 4).

### 4.3. Craniofacial Measurements

Craniofacial data for the Japanese population were obtained from both CBCT and MDCT images. CBCT images were acquired using a CBCT scanner with a voxel size of 0.3 mm (KaVo OP 3D Vision, Biberach, Germany), and MDCT images were obtained using a scanner with a voxel size of 2.0 mm (Aquilion Prime, Tochigi, Japan). For the Korean population, CBCT images were acquired using a scanner with a voxel size of 0.3 mm (Zenith 3D; Vatech Co., Seoul, Republic of Korea). For the Egyptian population, CBCT images were obtained using a scanner with a voxel size of 0.5 mm (Soredex SCANORA 3D; Nahkeantie 16, Tuusula, Finland). In this study, craniofacial images were obtained from three different CBCT scanners and one MDCT scanner, with voxel sizes ranging from 0.3 to 0.5 mm for CBCT and 2.0 mm for MDCT. Although the device type and voxel size differed across the populations, a previous study has reported that the variation in voxel size among CBCT devices has minimal impact on linear measurements and does not significantly affect measurement accuracy [42]. Moreover, measurement errors between CBCT and MDCT are within a negligible range in terms of measurement reliability [43]. In our previous study using the same dataset, we conducted validation using an aluminum block and confirmed that measurements were not affected by differences between CBCT devices or between CBCT and MDCT [44,45]. Based on these reports, we considered the image data acquired from different devices to be comparable in terms of measurement precision.

Cranial measurements were conducted by a single examiner at Kanagawa Dental University using both CBCT and MDCT images. Landmarks [19,46,47] were plotted and 3D coordinates were acquired using a 3D Slicer version 5.4.0 (Markups module, 3D Slicer) (Figure 1). Head orientation was standardized by aligning the midsagittal plane [48] with the nasion (n), anterior nasal spine (ans), and posterior nasal spine (pns), and aligning the horizontal plane with the Frankfurt horizontal plane (Transforms module, 3D Slicer version 5.4.0). After selecting the approximate landmark positions on the volume-rendered images, final adjustments were made using multiplanar reconstructions displaying axial, coronal, and sagittal views simultaneously. To assess measurement errors, 30 samples were randomly selected and measured again under the same conditions by the same examiner at least two weeks after the initial measurements. Measurement error was evaluated using Dahlberg’s formula [49].

To assess craniofacial morphological differences among the Japanese, Korean, and Egyptian populations, linear distances between landmark pairs were calculated from the 3D coordinates, resulting in 13 measurement variables. To evaluate associations between nasal root morphology and SNPs, seven angular measurements based on three-point landmarks were defined. One additional ratio variable was calculated based on the distance from the nasion to the mid-dacryon and the inter-dacryon distance. These comprised a total of eight variables characterizing nasal root morphology. To analyze shape variations in the upper face, a GPA was performed using only landmarks located in the upper facial region, followed by a PCA (Table S1). The decision to use only upper facial landmarks was based on the consideration that the major PC might otherwise be heavily influenced by variations in the mandible and cranial base, potentially obscuring the morphological characteristics of the nasal root region. To visualize the shape variation represented by each PC, wireframe models were generated (Figure 2 and Figure S1).

### 4.4. Statistical Analysis

To evaluate population differences in craniofacial morphology, group comparisons were performed using craniofacial measurement values. A one-way analysis of variance (ANOVA) followed by Tukey’s honestly significant difference test or a Kruskal–Wallis test followed by Dunn’s post hoc test were used as appropriate (Table 1 and Table 2).

To evaluate the associations between the eight nasal root morphology measurements and SNPs, multiple linear regression analyses were performed using a single-SNP model. The distributions of nasal root measurements were also compared among different genotypes of each SNP. For nasal root variables that exhibited significant associations with SNPs, further analyses were conducted using a multi-SNP model and a haplotype-based model. Associations between SNPs and upper face PC scores were also assessed using the single-SNP model (Table S2). In both the single- and multi-SNP models, the number of derived alleles was used as the explanatory variable, and in the multi-SNP model, an interaction term was included. In the haplotype model, the hGA haplotype was used as the reference, and the copy numbers (0 to 2) of hAG, hAA, and hGG were included as explanatory variables. Sex was included as a covariate in all models. To account for differences in genetic background among populations, multiple regression analyses were conducted individually for each population, and the results were integrated through meta-analysis using the inverse variance method. The results of a preliminary analysis examining the association between age and nasal root morphological measurements are presented in Table S3. Only a limited number of measurements showed statistically significant correlations, and the correlation coefficients were small. Including age in the statistical models did not affect the main results. Furthermore, nasal root growth is considered to be active during puberty and to almost cease in adulthood [50,51]. As this study included only adults aged 18 years or older, in whom skeletal growth is complete, age was not included in the final analytical models.

All statistical analyses were performed using RStudio: Integrated Development for R (RStudio, version 2024.12.0.467; PBC, Boston, MA, USA) and the Statistical Package for the Social Sciences (SPSS, version 28.0; IBM Corporation, Armonk, NY, USA). The statistical significance level was set at 5%.

## Figures and Tables

**Table 1:** Summary of craniofacial and nasal root morphology measurements for male individuals.

Male										
Category	Measurements	Japanese (*n* = 56)		Korean (*n* = 29)		Egyptian (*n* = 63)		Japanese vs. Korean	Japanese vs. Egyptian	Korean vs. Egyptian
		Mean	SD	Mean	SD	Mean	SD	*p*-Value	*p*-Value	*p*-Value
Craniofacial Morphology	n-me (mm)	126.03	5.92	128.73	5.96	122.15	6.48	0.14	**<0.01**	**<0.01**
	n-ans (mm)	56.25	3.08	57.48	2.23	55.13	5.92	0.14	**0.01**	**<0.01**
	a-ba (mm)	90.95	5.24	91.19	4.44	94.52	4.53	0.98	**<0.01**	**0.01**
	n-ba (mm)	102.54	4.53	104.06	5.17	104.46	4.26	0.32	0.06	0.92
	n-s (mm)	66.47	2.79	68.37	3.29	69.64	2.82	**0.01**	**<0.01**	0.13
	poR-poL (mm)	124.60	5.61	127.86	4.18	117.53	6.15	**0.03**	**<0.01**	**<0.01**
	sorR-sorL (mm)	59.54	3.29	62.51	2.72	56.98	3.98	**<0.01**	**<0.01**	**<0.01**
	fzsR-fzsL (mm)	104.84	4.94	108.14	3.74	105.44	4.81	**0.01**	0.99	**0.02**
	orR-orL (mm)	60.44	3.76	63.21	3.00	57.06	4.20	**0.02**	**<0.01**	**<0.01**
	dR-dL (mm)	19.36	1.99	20.44	2.07	20.07	2.29	0.16	0.40	0.99
	n-g (mm)	11.94	2.13	11.72	2.39	10.12	2.21	0.90	**<0.01**	**<0.01**
	n-rhi (mm)	26.28	3.96	28.35	2.37	24.23	3.18	**0.02**	**<0.01**	**<0.01**
	n-md (mm)	11.53	1.98	12.04	1.76	12.75	1.63	0.43	**<0.01**	0.18
Nasal Root Morphology	g-n-rhi (°)	138.54	7.95	139.58	5.89	126.38	9.01	0.99	**<0.01**	**<0.01**
	md-n-g (°)	150.69	8.36	150.70	7.70	142.39	7.20	0.99	**<0.01**	**<0.01**
	s-n-g (°)	113.43	5.83	112.76	5.11	116.47	6.27	0.87	**0.02**	**0.02**
	s-n-rhi (°)	107.95	6.66	107.58	5.48	117.05	6.57	0.97	**<0.01**	**<0.01**
	s-n-md (°)	37.45	6.75	38.42	6.57	26.13	6.16	0.79	**<0.01**	**<0.01**
	dR-n-dL (°)	80.71	10.88	81.06	8.60	76.55	7.11	0.98	**0.03**	0.07
	dR-s-dL (°)	18.97	2.07	19.47	1.90	19.44	2.33	0.57	0.46	0.99
	n-md/dR-dL ratio	0.60	0.11	0.59	0.09	0.64	0.08	0.93	0.09	0.09

Significant (*p* < 0.05).

**Table 2:** Summary of craniofacial and nasal root morphology measurements for female individuals.

Female										
Category	Measurements	Japanese (*n* = 145)		Korean (*n* = 45)		Egyptian (*n* = 79)		Japanese vs. Korean	Japanese vs. Egyptian	Korean vs. Egyptian
		Mean	SD	Mean	SD	Mean	SD	*p*-Value	*p*-Value	*p*-Value
Craniofacial Morphology	n-me (mm)	118.63	5.64	119.83	5.35	114.70	5.65	0.42	**<0.01**	**<0.01**
	n-ans (mm)	52.12	2.46	53.58	2.28	51.61	4.58	**0.01**	**0.04**	**<0.01**
	a-ba (mm)	85.86	4.52	86.26	4.42	90.37	5.08	0.87	**<0.01**	**<0.01**
	n-ba (mm)	96.53	4.13	98.10	3.99	99.29	4.19	0.07	**<0.01**	0.27
	n-s (mm)	62.64	2.74	64.04	2.93	66.14	2.95	**0.01**	**<0.01**	**<0.01**
	poR-poL (mm)	117.18	4.84	120.53	4.87	110.59	5.08	**<0.01**	**<0.01**	**<0.01**
	sorR-sorL (mm)	57.69	2.80	59.13	2.76	55.23	3.36	**0.01**	**<0.01**	**<0.01**
	fzsR-fzsL (mm)	99.88	3.53	101.26	3.74	101.77	4.29	0.06	**<0.01**	0.99
	orR-orL (mm)	57.91	3.59	59.19	2.31	54.68	3.84	0.15	**<0.01**	**<0.01**
	dR-dL (mm)	18.59	1.86	19.26	2.02	19.49	1.77	0.29	**0.01**	0.99
	n-g (mm)	13.96	3.14	13.23	2.42	11.80	3.26	0.90	**<0.01**	**0.02**
	n-rhi (mm)	24.95	2.85	26.98	2.82	24.08	3.39	**<0.01**	0.10	**<0.01**
	n-md (mm)	10.43	1.48	10.68	1.62	11.63	1.66	0.61	**<0.01**	**<0.01**
Nasal Root Morphology	g-n-rhi (°)	147.84	5.11	149.28	5.60	141.36	5.79	0.26	**<0.01**	**<0.01**
	md-n-g (°)	147.00	6.20	147.60	7.71	136.07	8.63	0.88	**<0.01**	**<0.01**
	s-n-g (°)	106.16	4.40	104.93	5.83	105.24	5.46	0.32	0.38	0.94
	s-n-rhi (°)	105.93	5.19	105.73	5.70	113.26	5.16	0.97	**<0.01**	**<0.01**
	s-n-md (°)	41.02	5.29	43.03	5.49	31.03	6.90	0.11	**<0.01**	**<0.01**
	dR-n-dL (°)	83.75	8.13	84.43	8.33	80.38	8.08	0.88	**0.01**	**0.02**
	dR-s-dL (°)	19.09	1.94	19.26	2.29	19.52	1.90	0.99	0.71	0.74
	n-md/dR-dL ratio	0.56	0.08	0.56	0.08	0.60	0.09	0.89	**0.01**	**0.02**

Significant (*p* < 0.05).

**Table 3:** Allele frequencies and LD coefficients of *PAX3* SNPs.

rs Number	Chr: Position	Location	Alleles		Derived Allele Frequency			LD Coefficients (D’/r^2^)		
			Ancestral	Derived	Japanese	Korean	Egyptian	Japanese	Korean	Egyptian
rs9288572	chr2:222097644	Intergenic	G	A	0.572	0.561	0.197	0.181/0.019	0.019/0.028	0.063/0.001
rs7559271	chr2:222203567	Intronic	A	G	0.701	0.649	0.532			

LD, linkage disequilibrium.

**Table 4:** Haplotypes for *PAX3* variants.

Haplotype	Haplotype Frequency		
	Japanese	Korean	Egyptian
hAG	0.419	0.384	0.108
hAA	0.153	0.177	0.089
hGG	0.283	0.265	0.424
hGA	0.145	0.174	0.379

**Table 5:** Associations between *PAX3* SNPs and nasal root measurements based on multiple regression analysis using a single-SNP model.

Measurements	Japanese (*n* = 201)			Korean (*n* = 74)			Egyptian (*n* = 142)			Combined		
	B	SD	*p*-Value	B	SD	*p*-Value	B	SD	*p*-Value	B	SD	*p*-Value
**rs9288572**												
g-n-rhi	1.090	0.606	7.5 × 10^−2^	0.426	0.894	6.4 × 10^−1^	0.950	1.12	4.0 × 10^−1^	0.892	0.458	5.1 × 10^−2^
md-n-g	−1.621	0.684	**1.9 × 10^−2^**	0.616	1.197	6.1 × 10^−1^	−0.470	1.216	7.0 × 10^−1^	−0.955	0.534	7.4 × 10^−2^
s-n-g	−0.784	0.485	1.1 × 10^−1^	0.833	0.859	3.4 × 10^−1^	−0.010	0.884	9.9 × 10^−1^	−0.322	0.381	4.0 × 10^−1^
s-n-rhi	−0.260	0.571	6.5 × 10^−1^	−1.270	0.859	1.4 × 10^−1^	−0.992	0.875	2.6 × 10^−1^	−0.666	0.418	1.1 × 10^−1^
s-n-md	−0.799	0.576	1.7 × 10^−1^	−0.242	0.923	7.9 × 10^−1^	−0.365	0.997	7.2 × 10^−1^	−0.589	0.439	1.8 × 10^−1^
dR-n-dL	0.468	0.908	6.1 × 10^−1^	−1.265	1.304	3.4 × 10^−1^	1.560	1.15	1.8 × 10^−1^	0.389	0.625	5.3 × 10^−1^
dR-s-dL	−0.111	0.199	5.8 × 10^−1^	−0.469	0.329	1.6 × 10^−1^	0.288	0.318	3.7 × 10^−1^	−0.097	0.150	5.2 × 10^−1^
n-md/dR-dL	−0.003	0.009	7.3 × 10^−1^	0.014	0.013	3.0 × 10^−1^	−0.017	0.013	1.8 × 10^−1^	−0.002	0.006	7.2 × 10^−1^
**rs7559271**												
g-n-rhi	0.258	0.675	7.0 × 10^−1^	−0.440	0.994	6.6 × 10^−1^	0.720	0.815	3.8 × 10^−1^	0.256	0.461	5.8 × 10^−1^
md-n-g	−1.878	0.758	**1.4 × 10^−2^**	1.660	1.328	2.2 × 10^−1^	−2.469	0.863	**5.0 × 10^−3^**	−1.546	0.523	**3.1 × 10^−3^**
s-n-g	−1.343	0.533	**1.3 × 10^−2^**	1.930	0.941	**4.4 × 10^−2^**	−1.941	0.624	**2.0 × 10^−3^**	−1.044	0.372	**5.0 × 10^−3^**
s-n-rhi	1.090	0.629	8.4 × 10^−2^	−1.469	0.963	1.3 × 10^−1^	1.214	0.636	5.8 × 10^−2^	0.686	0.406	9.1 × 10^−2^
s-n-md	−0.519	0.641	4.2 × 10^−1^	−0.354	1.032	7.3 × 10^−1^	−0.504	0.727	4.9 × 10^−1^	−0.484	0.436	2.7 × 10^−1^
dR-n-dL	1.836	0.997	6.7 × 10^−2^	0.601	1.467	6.8 × 10^−1^	0.270	0.848	7.5 × 10^−1^	0.874	0.591	1.4 × 10^−1^
dR-s-dL	0.114	0.221	6.1 × 10^−1^	0.557	0.368	1.3 × 10^−1^	−0.217	0.232	3.5 × 10^−1^	0.052	0.147	7.2 × 10^−1^
n-md/dR-dL	−0.019	0.010	6.4 × 10^−2^	−0.005	0.015	7.6 × 10^−1^	−0.002	0.009	8.3 × 10^−1^	−0.009	0.006	1.5 × 10^−1^

Significant (*p* < 0.05). The number of derived alleles (0–2) was used as an explanatory variable, with sex included as a covariate. B, regression coefficient; SD, standard deviation.

**Table 6:** Differences in nasal root measurements across *PAX3* SNP genotypes.

Measurements	Group	Genotype			*p*-Value
		AA	AG	GG	
**rs7559271**					
md-n-g	Japanese	151.44 ± 8.16	148.64 ± 7.41	146.90 ± 6.31	**0.031**
	Korean	147.69 ± 3.86	147.75 ± 7.68	150.32 ± 8.69	0.378
	Egyptian	141.14 ± 8.03	139.70 ± 7.92	136.00 ± 9.25	**0.015**
s-n-g	Japanese	111.88 ± 6.17	108.3 6± 5.73	107.42 ± 5.67	**0.016**
	Korean	106.25 ± 5.15	106.94 ± 5.92	109.67 ± 7.72	0.188
	Egyptian	112.46 ± 8.70	110.50 ± 7.41	108.06 ± 8.00	**0.044**

Significant (*p* < 0.05).

**Table 7:** Associations between *PAX3* SNP haplotypes and nasal root measurements based on multiple regression analysis in the haplotype model.

Measurements	Explanatory Variable	Japanese (*n* = 201)			Korean (*n* = 74)			Egyptian (*n* = 142)			Combined		
		B	SD	*p*-Value	B	SD	*p*-Value	B	SD	*p*-Value	B	SD	*p*-Value
md-n-g	hAG	−3.296	0.968	**8.1 × 10^−4^**	1.127	2.137	6.0 × 10^−1^	−2.974	1.553	5.8 × 10^−2^	−2.648	0.767	**5.5 × 10^−4^**
	hAA	−2.643	1.291	**4.2 × 10^−2^**	−0.537	2.380	8.2 × 10^−1^	−0.104	1.782	9.5 × 10^−1^	−1.570	0.957	1.0 × 10^−1^
	hGG	−2.496	1.063	**2.0 × 10^−2^**	1.491	2.183	5.0 × 10^−1^	−2.379	0.948	**1.3 × 10^−2^**	−2.058	0.673	**2.2 × 10^−3^**
s-n-g	hAG	−1.702	0.686	**1.4 × 10^−2^**	1.984	1.493	1.9 × 10^−1^	−2.180	1.121	5.4 × 10^−2^	−1.324	0.545	**1.5 × 10^−2^**
	hAA	0.282	0.914	7.6 × 10^−1^	1.147	1.663	4.9 × 10^−1^	0.765	1.287	5.5 × 10^−1^	0.561	0.680	4.1 × 10^−1^
	hGG	−0.628	0.753	4.1 × 10^−1^	3.292	1.525	**3.4 × 10^−2^**	−1.737	0.685	**1.2 × 10^−2^**	−0.785	0.481	1.0 × 10^−1^

Significant (*p* < 0.05). The hGA haplotype was used as the reference, and the copy numbers (0 to 2) of hAG, hAA, and hGG were included as explanatory variables, with sex included as a covariate. B, regression coefficient; SD, standard deviation.

**Table 8:** Associations between *PAX3* SNPs and nasal root measurements based on multiple regression analysis in a multi-SNP model.

Measurements	Explanatory Variable	Japanese (*n* = 201)			Korean (*n* = 74)			Egyptian (*n* = 142)			Combined		
		B	SD	*p*-Value	B	SD	*p*-Value	B	SD	*p*-Value	B	SD	*p*-Value
md-n-g	rs9288572	−2.922	1.631	7.5 × 10^−2^	0.087	2.914	9.8 × 10^−1^	0.787	2.005	7.0 × 10^−1^	−1.202	1.161	3.0 × 10^−1^
	rs7559271	−2.893	1.429	**4.4 × 10^−2^**	1.375	2.234	5.4 × 10^−1^	−2.075	1.017	**4.3 × 10^−2^**	−1.900	0.777	**1.4 × 10^−2^**
	Interaction	1.101	1.078	3.1 × 10^−1^	0.225	1.819	9.0 × 10^−1^	−1.115	1.524	4.7 × 10^−1^	0.336	0.792	6.7 × 10^−1^
s-n-g	rs9288572	0.080	1.158	9.5 × 10^−1^	−0.847	2.049	6.8 × 10^−1^	0.452	1.453	7.6 × 10^−1^	0.049	0.828	9.5 × 10^−1^
	rs7559271	−0.669	1.014	5.1 × 10^−1^	0.866	1.571	5.8 × 10^−1^	−1.809	0.736	**1.5 × 10^−2^**	−1.129	0.557	**4.3 × 10^−2^**
	Interaction	−0.513	0.765	5.0 × 10^−1^	0.994	1.279	4.4 × 10^−1^	−0.380	1.104	7.3 × 10^−1^	−0.185	0.564	7.4 × 10^−1^

Significant (*p* < 0.05). The number of derived alleles (0–2) was used as an explanatory variable, with sex included as a covariate. The interaction term between the two SNPs was also included as an explanatory variable. B, regression coefficient; SD, standard deviation.

**Table 9:** Associations between *PAX3* SNPs and upper face principal components based on multiple regression analysis.

Outcome Variable	Explanatory Variable	Japanese (*n* = 201)			Korean (*n* = 74)			Egyptian (*n* = 142)			Combined		
		B	SD	*p*-Value	B	SD	*p*-Value	B	SD	*p*-Value	B	SD	*p*-Value
PC1	rs7559271	0.000	0.002	0.868	0.008	0.003	**0.016**	−0.001	0.002	0.538	0.001	0.001	0.414
PC5	rs7559271	−0.005	0.002	**0.048**	0.001	0.003	0.656	0.004	0.002	**0.029**	−0.001	0.001	0.859

Significant (*p* < 0.05). The number of derived alleles (0–2) was used as an explanatory variable, with sex included as a covariate. B, regression coefficient; SD, standard deviation.

## Data Availability

The data presented in this study are available from the corresponding author upon request.

## Supplementary Materials

The following supporting information can be downloaded at: https://www.mdpi.com/article/10.3390/ijms26167842/s1.

## Abbreviations

The following abbreviations are used in this manuscript:

*PAX3*	Paired box gene 3
WS	Waardenburg syndrome
CDHS	Craniofacial-deafness-hand syndrome
GWAS	Genome-Wide Association Study
SNP	Single-nucleotide polymorphism
CBCT	Cone-beam computed tomography
MDCT	Multi-detector computed tomography
LD	Linkage disequilibrium
PCA	Principal component analysis
PC	Principal component
GPA	Generalized Procrustes analysis
