# Supplementary material for: Association of PAX3 Gene Polymorphism with Three-Dimensional Nasal Root Morphology
Source: Int J Mol Sci. 2025 Aug 14;26(16):7842. doi: 10.3390/ijms26167842 (PMC12386190; doi:10.3390/ijms26167842)
Supplement: Supplementary file 1 [file ijms-26-07842-s001.zip › ijms-3782038-supplementary.pdf]

Supplementary Information

Association of *PAX3* Gene Polymorphism with Three-Dimensional Nasal Root Morphology

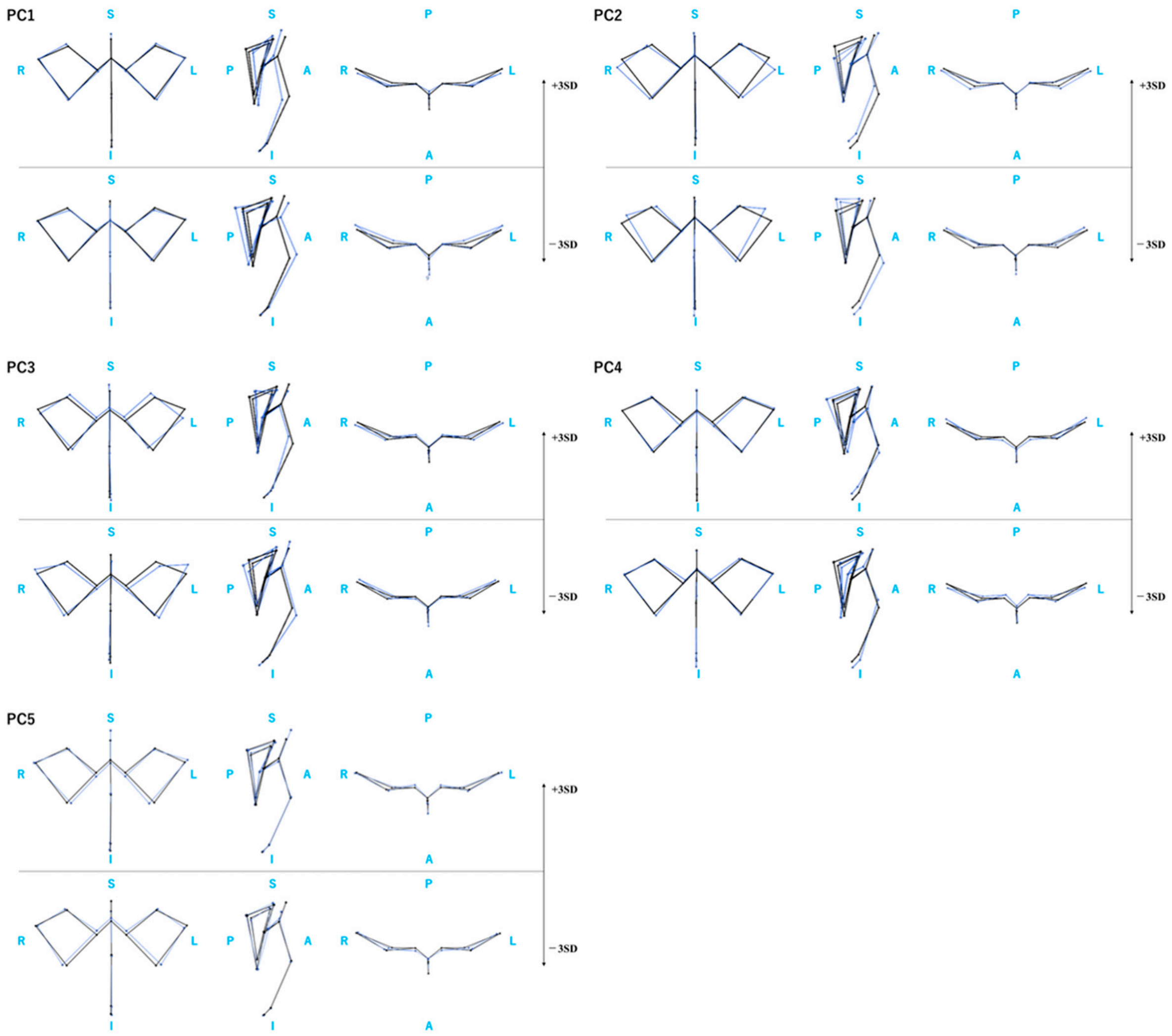

Figure S1. Shape variation along PC1 to PC5 visualized as deviations of ±3 SD from the mean 3D coordinates. S—superior, I—inferior, A—anterior, P—posterior, R—right, L—left. black lines represent the average configuration, while blue lines represent the variation of interest.

Table S1. Eigenvalues in the principal component (PC) analysis for the upper face.

	Eigenvalue	Contribution	Cumulative contribution
PC1	0.042	26.07%	26.07%
PC2	0.031	14.35%	40.42%
PC3	0.027	10.78%	51.20%
PC4	0.022	6.94%	58.14%
PC5	0.020	5.73%	63.87%

Table S2. Associations between *PAX3* SNPs and upper-face principal components based on multiple regression analysis.

Outcome variable	Japanese (n=201)			Korean (n=74)			Egyptian (n=142)			Combined		
	B	SD	P-value	B	SD	P-value	B	SD	P-value	B	SD	P-value
**rs9288572**												
PC1	0.001	0.002	0.468	0.002	0.003	0.550	0.003	0.003	0.412	0.002	0.001	0.241
PC2	-0.002	0.002	0.542	-0.001	0.003	0.688	0.008	0.006	0.191	-0.001	0.002	0.533
PC3	-0.001	0.002	0.595	0.001	0.003	0.584	0.000	0.005	0.929	0.000	0.002	0.826
PC4	0.000	0.002	0.984	-0.004	0.003	0.169	-0.004	0.003	0.225	-0.002	0.001	0.191
PC5	-0.003	0.002	0.104	0.001	0.002	0.760	-0.001	0.003	0.629	-0.001	0.001	0.434
**rs7559271**												
PC1	0.000	0.002	0.868	0.008	0.003	**0.016**	-0.001	0.002	0.538	0.001	0.001	0.414
PC2	0.002	0.003	0.495	-0.002	0.004	0.555	-0.004	0.005	0.342	0.000	0.002	0.892
PC3	-0.001	0.003	0.823	-0.001	0.003	0.827	0.000	0.004	0.971	-0.001	0.002	0.677
PC4	0.000	0.002	0.945	-0.005	0.003	0.082	0.004	0.002	0.136	0.001	0.001	0.570
PC5	-0.005	0.002	**0.048**	0.001	0.003	0.656	0.004	0.002	**0.029**	0.000	0.001	0.859

Significant ($p < 0.05$).

The number of derived alleles (0–2) was used as an explanatory variable, with sex included as a covariate.
B, regression coefficient; SD, standard deviation.

Table S3. Correlation between age and nasal root morphological measurements based on Pearson's or Spearman's correlation analysis.

**Measurements**	**Japanese**		**Korean**		**Egyptian**	
	**r**	**P-value**	**r**	**P-value**	**r**	**P-value**
g-n-rh	-0.116	0.100	0.030	0.802	-0.169	**0.044**
md-n-g	0.058	0.414	0.152	0.195	0.070	0.410
s-n-g	0.032	0.648	0.024	0.839	0.084	0.322
s-n-rh	0.083	0.240	-0.065	0.583	0.040	0.637
s-n-md	0.037	0.606	0.155	0.187	-0.012	0.885
dR-n-dL	-0.004	0.955	0.066	0.578	-0.009	0.920
dR-s-dL	0.000	0.998	0.064	0.590	0.228	**0.006**
n-md/dR-dL	0.037	0.605	-0.054	0.649	-0.010	0.910

Significant ($p < 0.05$).
